# Reliability and Construct Validity of the Six‐Minute Step Test in Individuals With Non‐Specific Chronic Low Back Pain

**DOI:** 10.1002/pri.70295

**Published:** 2026-07-17

**Authors:** Francisco Basílio da Silva‐Júnior, ⁠André Pontes‐Silva, ⁠Yane Pelicer‐Marques, Sabrina Marinho Coutinho, Marco Fabrício Dias‐Peixoto, Kassiana de Araujo Pessôa, Andréa Dias Reis, Cristiano Teixeira Mostarda, Almir Vieira Dibai‐Filho, Flávio de Oliveira Pires

**Affiliations:** ^1^ Federal University of Maranhão São Luís Maranhão Brazil; ^2^ School of Public Health University of São Paulo São Paulo Brazil; ^3^ Departament of Physical Education Federal University of the Jequitinhonha and Mucuri Valleys Diamantina Minas Gerais Brazil

**Keywords:** 6MST, chronic pain, musculoskeletal system, spine

## Abstract

**Background and Purpose:**

The six‐minute step test (6MST) is a practical, field‐based assessment that may provide useful information about stepping performance in individuals with non‐specific chronic low back pain (NSCLBP). However, evidence regarding its measurement properties in this population is limited. The aim of this study was to evaluate the inter‐rater reliability and construct validity of the 6MST in individuals with NSCLBP.

**Methods:**

We conducted a cross‐sectional psychometric study to evaluate the 6MST's inter‐rater reliability and construct validity using a known‐groups approach. Thirty‐one individuals with NSCLBP and 31 age‐ and sex‐matched healthy controls completed the 6MST. Two blinded raters administered the test independently on separate occasions 7 days apart. We assessed inter‐rater reliability using the intraclass correlation coefficient (ICC), standard error of measurement (SEM), minimal detectable change (MDC), and Bland–Altman analysis. Construct validity was examined by comparing 6MST performance between groups using hypothesis testing and effect sizes (Cohen's d). Secondary outcomes included heart rate responses, perceived exertion, pain intensity, disability, pain catastrophizing, and kinesiophobia.

**Results:**

The 6MST demonstrated excellent inter‐rater reliability, with ICCs of 0.915 (95% CI: 0.83–0.96) for the NSCLBP group and 0.973 (95% CI: 0.94–0.99) for controls. SEM and MDC values were 6 and 17 steps in the NSCLBP group and 3 and 8 steps in the controls, respectively. Bland–Altman analyses revealed good agreement and no evidence of proportional bias. Construct validity was supported by significantly lower performance in the NSCLBP group compared with the control group (mean differences: 37 and 33 steps for raters 1 and 2, respectively, both with *p* values less than 0.001), with very large effect sizes (Cohen's *d* = 1.91 and 1.76). Individuals with NSCLBP showed lower attainment of the predefined target heart rate and greater increases in pain intensity following the test, despite similar perceived exertion.

**Discussion:**

The 6MST demonstrated excellent inter‐rater reliability and satisfactory construct validity for assessing stepping performance in individuals with NSCLBP. Compared with healthy controls, individuals with NSCLBP completed fewer steps, were less likely to reach the predefined target heart rate during testing, and reported greater increases in pain intensity despite similar perceived exertion. These findings support the use of the 6MST as a practical, low‐cost, performance‐based assessment of stepping performance in this population. However, the observed heart rate responses should not be interpreted as evidence of differences in exercise tolerance, aerobic capacity, or cardiopulmonary function.

## 1 Introduction

Globally, non‐specific chronic low back pain (NSCLBP) is the leading cause of years lived with disability (Ferreira et al. 2023). It is defined as pain localized between the lower rib margin and the inferior gluteal folds that persists for more than 3 months, with or without radiation to the lower limbs (Trinidad‐Fernández et al. 2021). When no specific anatomical or pathological cause is identified through clinical or imaging assessments, the condition is classified as NSCLBP. NSCLBP accounts for up to 90% of all cases (Burin and Mansur Guedes 2023).

NSCLBP is a major public health concern due to its association with functional disability, work absenteeism, increased healthcare utilization, and a substantial socioeconomic burden worldwide (Wu et al. 2020). Epidemiological data indicate an increasing prevalence of NSCLBP between 1999 and 2021, especially among individuals of working age (15–65 years) (Ferreira et al. 2023).

NSCLBP is a multifactorial and complex condition involving biological, psychological, and social determinants, making it a frequent subject of investigation (Kehl et al. 2024; Lee et al. 2015; Giesecke et al. 2004). Systematic reviews have shown that this complexity poses challenges to the development of effective therapeutic strategies, often resulting in inconsistencies and uncertainties across clinical guidelines. These challenges may contribute to persistent pain and long‐term disability (Gianola et al. 2022; Barreto et al. 2021).

In clinical practice, field‐based or submaximal tests are commonly used to evaluate physical performance under standardized conditions. These tests are cost‐effective, easy to administer, and adaptable to different settings and populations. They may also provide clinically meaningful information. Because they resemble activities commonly performed in daily life, these tests may better reflect real‐world functional demands than laboratory‐based maximal exercise protocols while remaining practical (De Jesus et al. 2022).

Denteneer et al. conducted a systematic review and identified only two studies investigating the 6MST in individuals with NSCLBP (Denteneer et al. 2018). Neither study established the test's reliability or validity. The test was performed using a standardized step platform (20 cm high, 50 cm wide, and 25 cm deep) with a non‐slip surface. Participants are instructed to step up and down as quickly as possible for 6 minutes at their own pace without using their arms for support. Verbal encouragement follows the standardized protocol of the six‐minute walk test. The outcome measure is the total number of steps completed during the test (Ritt et al. 2021).

Emerging evidence suggests that impairments in hip mobility, including stiffness and instability, may co‐exist with NSCLBP and be associated with altered movement strategies during functional tasks (Rosa and Felício 2022). However, the relationship between these impairments and performance on the 6MST has not been directly established. From a theoretical perspective, stepping activities require coordinated lower‐limb and lumbopelvic control. This provides a rationale for investigating whether this performance‐based test can identify limitations in this population (Salles Albuquerque et al. 2022).

Previous studies have demonstrated the adequate measurement properties of shorter step tests, such as the two‐minute step test. However, these findings cannot be directly extrapolated to the 6MST. Differences in test duration substantially influence physiological demands, fatigue development, pacing strategies, and stepping performance. The six‐minute protocol requires sustained repetitive stepping over a prolonged period and may challenge lower‐limb endurance and pacing strategies during task execution, which could provide a more comprehensive assessment of activity limitations relevant to individuals with NSCLBP. Thus, validating the reliability and validity of the 6MST is an important step toward expanding the range of available performance‐based outcome measures for this population and helping clinicians select assessment tools that more accurately reflect the prolonged functional activities encountered in daily life.

Unlike the six‐minute walk test, which primarily evaluates horizontal locomotion, the 6MST incorporates repetitive vertical displacement of body mass and requires continuous concentric and eccentric activation of the lower‐limb musculature while challenging lumbopelvic control. Consequently, it may be particularly useful in environments where walking space is limited and in situations where clinicians seek a compact performance‐based test capable of reproducing functional tasks involving stair negotiation or repeated stepping. Establishing its measurement properties in individuals with NSCLBP therefore expands the range of validated assessment tools available for clinical practice rather than replacing existing measures.

This study is justified by the 6MST's potential to provide a practical assessment of stepping performance in individuals with NSCLBP, particularly in activities that require increased load on the lower limbs and lumbopelvic region. These insights could expand the range of performance‐based assessments available for individuals with NSCLBP. Thus, this study aimed to evaluate the inter‐rater reliability of the 6MST in assessing stepping performance in individuals with NSCLBP.

## 2 Methods

### 2.1 Study Design and Ethical Considerations

The reliability and construct validity of the 6MST study are based on a hypothesis‐testing approach that uses a control group, as well as the Guidelines for Reporting Reliability and Agreement Studies (Kottner et al. 2011). The study was approved by the Research Ethics Committee of the Universidade Federal do Maranhão (report number 5404643).

Data collection was conducted from 2023 to 2024 at the Universidade Federal do Maranhão. Two of the five trained raters were randomly selected and blinded to each other's assessments and questionnaire results. They were designated as raters 1 and 2. Only rater 1 had access to the questionnaire data. Both raters administered the 6MST independently and without observing each other's procedures. All participants received detailed information about the study procedures and provided written informed consent prior to participation.

### 2.2 Sample Size, Inclusion, and Exclusion Criteria

The sample size calculation for the reliability analysis indicated a minimum of 31 individuals based on intraclass correlation coefficients of 0.750 and 0.903 (the minimum acceptable and expected reliability, respectively) with a 5% significance level and 80% statistical power. This calculation was performed using an online tool (Wan Nor Arifin: https://wnarifin.github.io/ssc/ssicc.html). This calculation was specifically for estimating the intraclass correlation coefficient in the reliability analysis and was not based on hypotheses related to construct validity or between‐group comparisons.

Participants were recruited through social media (Instagram and WhatsApp) and printed advertisements distributed on the campus. The sample was divided into two groups: those with NSCLBP and a control group. The inclusion criteria were as follows: adults aged 18–45 years of either sex who had reported NSCLBP for more than three months and who had not received a medical diagnosis of discopathy, lumbosacral disc herniation, or other specific causes of low back pain. The age range was selected to reduce heterogeneity related to age‐associated declines in physical performance, multimorbidity, and degenerative musculoskeletal conditions, thereby improving the internal validity of this initial psychometric evaluation.

Non‐inclusion criteria: presence of conditions that could interfere with safe completion of the test protocol, including pulmonary or cardiovascular diseases (except controlled hypertension without beta‐blocker use) (Salles Albuquerque et al. 2022) musculoskeletal dysfunction of the lumbar spine, hip, or lower limbs due to prior surgery or autoimmune rheumatic or neurological diseases; visual impairment; hemodynamic instability at rest; or pregnancy (Oliveira et al. 2021). Exclusion criteria: positive Slump or straight leg raise (Lasègue) test (Berthelot et al. 2021) abnormal neuromuscular reflexes (patellar or Achilles reflexes); inability to complete the test due to discomfort (e.g., dizziness or pain); absence from the second assessment; or failure to comply with pre‐test instructions (Pontes‐Silva et al. 2021).

### 2.3 Pre‐Assessment Instructions

Individuals were instructed to abstain from alcohol, caffeine, and vigorous physical activity for 24 h before testing. They were also instructed to avoid eating for at least 2 hours before the assessment. They were also required to wear appropriate clothing and footwear for physical activity.

### 2.4 Procedures

Assessments were conducted on two occasions, 7 days apart. This interval was chosen to minimize immediate recall of test performance while reducing the likelihood of clinically significant changes in functional status. We acknowledge that this design simultaneously varied examiner and assessment occasion, which may have introduced familiarization or adaptation effects in addition to inter‐rater variability. Rater 1 performed the first assessment, and rater 2 conducted the second. Each individual was evaluated once by each rater.

To ensure blinding, the rater recorded the results independently and only merged the datasets after completing data collection. During the first session, Rater 1 applied the eligibility criteria, obtained informed consent, performed the neurological screening, and administered the questionnaires (without accessing their results). Rater 2 repeated only the 6MST, using the same protocol. Participants were instructed not to discuss their previous assessment, questionnaire responses, or test performance with the second examiner. Similarly, the raters had no access to each other's records until the end of the data collection period, thereby preserving assessor blinding throughout the study (Pontes‐Silva et al. 2021).

### 2.5 Clinical and Physiological Assessments

Pain assessment: Pain intensity was measured using the Numeric Pain Rating Scale prior to neurological testing to avoid bias due to provoked pain. This validated scale ranges from 0 (“no pain”) to 10 (“worst imaginable pain”) (Ferreira‐Valente et al. 2011). Hemodynamic assessment: Resting heart rate and blood pressure were measured using an automated monitor (Incoterm, model MB050). Heart rate was continuously monitored during testing using a chest strap (Polar Electro H10, Finland). Blood pressure was assessed at rest and immediately after the test (Penha et al. 2023).

Anthropometry: stature and body mass were measured using a calibrated scale (Welmy, model W200 A). Body mass index was calculated (kg/m^2^) according to national guidelines. Neurological screening: Slump and straight leg raise tests were used to identify signs of nerve root involvement. A positive result was defined as low back pain with or without radiation along the sciatic nerve. Patellar and Achilles reflexes were assessed using a reflex hammer (BIC, model Buck) and classified as absent, reduced, normal, or exaggerated. Positive findings led to exclusion due to suspicion of specific low back pain (Pontes‐Silva et al. 2022).

### 2.6 Questionnaires

All questionnaires were completed electronically via a supervised online form.

- Roland‐Morris Disability Questionnaire: A 24‐item instrument (score range: 0–24), with higher scores indicating greater disability (Nusbaum et al. 2001).
- Tampa Scale for Kinesiophobia: A 17‐item questionnaire scored on a 4‐point Likert scale, with total scores ranging from 17 to 68 (Siqueira et al. 2007).
- Pain‐Related Catastrophizing Thoughts Scale: A 9‐item instrument scored from 0 to 5, with higher scores indicating greater catastrophizing (Sardá Junior et al. 2008).
- International Physical Activity Questionnaire (short version): Assesses physical activity level over the past 7 days (Matsudo et al).
- Borg CR10 Scale: Used to assess perceived exertion every 2 min during the test (Gunnar 1998).

### 2.7 Six‐Minute Step Test

The test was performed using a standardized step (20 cm stature, 50 cm width, 25 cm depth) with a non‐slip surface (Ritt et al. 2021). Individuals were instructed to step up and down as many times as possible within 6 minutes at a self‐selected pace, without upper limb support. Heart rate was continuously monitored, and maximum heart rate was estimated using the equation: 220 − age (Shookster et al. 2020). A threshold of 85% of maximum heart rate was adopted for safety monitoring.

Individuals were allowed to slow down, pause, or rest as needed; however, the timer was not stopped. Standardized verbal encouragement was provided at each minute, following the protocol of the six‐minute walk test (Da Costa et al. 2017). No familiarization trial was conducted. The primary outcome was the total number of steps completed.

### 2.8 Statistical Analysis

Statistical analyses were performed using JAMOVI (version 2.3.17), except for the Bland–Altman plots, which were generated using MedCalc (version 23.0.9). Data normality was assessed using the Shapiro–Wilk test. Continuous variables are presented as the mean and standard deviation, and categorical variables are presented as frequencies. A 5% significance level (*p* < 0.05) was adopted for all two‐tailed tests.

We assessed inter‐rater reliability using the intraclass correlation coefficient (ICC2,1; two‐way random‐effects model, absolute agreement). The standard error of measurement and minimal detectable change were calculated according to established formulas (Weir 2005). Reliability thresholds followed the established criteria of poor (< 0.40), moderate (0.40–0.74), good (0.75–0.90), and excellent (> 0.90) (De Jesus et al. 2022). Confidence interval interpretation followed Kool's recommendations (Koo and Li 2016).

The agreement between raters was further analyzed using Bland–Altman plots. The mean bias and 95% limits of agreement (mean difference ± 1.96 × standard deviation) were calculated (Bland and Altman 1986; Sandberg et al. 2020). Construct validity was evaluated using a hypothesis‐testing approach by comparing known groups (control vs. individuals with non‐specific chronic low back pain [NSCLBP]). After confirming the assumptions of normality (Shapiro–Wilk test) and homoscedasticity (Levene's test), independent *t*‐tests were applied. When the assumptions were not met, the Mann–Whitney test was used (Souza et al. 2017).

The effect size was calculated using Cohen's *d* to assess clinical relevance (Lakens 2013), with values interpreted as small (0.2), medium (0.5), or large (0.8). Associations between independent variables and test performance were analyzed using Pearson or Spearman correlation coefficients according to the data distribution. The strength of the correlation was interpreted as weak (0–0.20), moderate (0.21–0.50), strong (0.51–0.80), or very strong (0.81–1.00) (De Jesus et al. 2022).

## 3 Results

**FIGURE 1:**
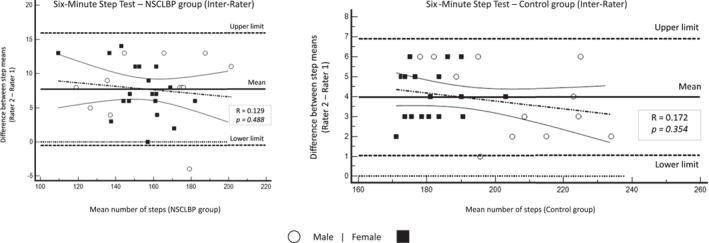
Bland–Altman Plots of Inter‐Rater Agreement for the Six‐Minute Step Test in Individuals with Non‐Specific Chronic Low Back Pain and Controls. Bland–Altman plots illustrating inter‐rater agreement for the six‐minute step test in the non‐specific chronic low back pain group (left panel) and control group (right panel). The x‐axis represents the mean number of steps obtained from both raters, while the y‐axis shows the difference between raters (Rater 2 − Rater 1). The solid horizontal line indicates the mean bias, and the dashed lines represent the limits of agreement (mean ± 1.96 standard deviations). The dotted line represents the trend of differences across measurements. Symbols distinguish sex (open circles: male; filled squares: female). Correlation coefficients (R) and *p*‐values are displayed to assess proportional bias.

A total of 55 individuals with NSCLBP and 40 healthy individuals were initially considered eligible. After applying the exclusion criteria, 43 (78%) individuals with NSCLBP and 31 (78%) healthy individuals remained. To improve group comparability and reduce bias, the NSCLBP group was randomized and matched to the control group by sample size and sex distribution, resulting in 31 individuals per group, with 61% females (*n* = 19) and 39% males (*n* = 12) in both groups (Figure 1).

Anthropometric characteristics are presented in Table 1 as mean ± standard deviation. Individuals ranged in age from 18 to 42 years. Mean age varied between 27 ± 6 years (female NSCLBP group) and 30 ± 9 years (female control group). Mean age was comparable between groups, although female participants in the control group were slightly older than those in the NSCLBP group (*p* = 0.041).

**TABLE 1:** Individual characteristics.

Variable	CLBP (*n* = 31)	Control (*n* = 31)
Age (years)	28 ± 7^*^	29 ± 8
Stature (m)	1.67 ± 0.10^*^	1.65 ± 0.10^*^
Body mass (kg)	73.8 ± 12.7	64.6 ± 9.1^*^
BMI (kg/m^2^)	26.4 ± 3.5^*^	23.9 ± 2.8^*^
BMI classification, *n* (%)		
Normal weight	11 (35.5)	11 (35.5)
Overweight	16 (51.6)	20 (64.5)
Obesity	4 (12.9)	
Physical activity level (IPAQ), *n* (%)		
Active	14 (45.2)	13 (41.9)
Sedentary	17 (54.8)	18 (58.1)

Abbreviations: BMI: body mass index; CLBP: non‐specific chronic low back pain group; IPAQ: International Physical Activity Questionnaire; SD: standard deviation.

^*^ *p* > 0.05 in the Shapiro–Wilk test, indicating normal distribution.

Stature values were similar across groups, ranging from 1.59 ± 0.1 m (female control group) to 1.73 ± 0.1 m (male control group). Body mass ranged from 61.1 ± 5.4 kg (female control group) to 80.5 ± 14.4 kg (male NSCLBP group). Overall, individuals with NSCLBP had a higher body mass (73.8 ± 12.7 kg) compared to controls (64.6 ± 9.1 kg), with a statistically significant difference observed only among females (*p* = 0.037).

Body mass index indicated a high prevalence of overweight in both groups, affecting more than 50% of individuals (NSCLBP group: 51.6%; control group: 64.5%). Obesity was observed in 13% of individuals in the NSCLBP group. Physical activity levels assessed by the International Physical Activity Questionnaire were similar between groups, with comparable proportions of active (NSCLBP group: 45.2%; control group: 41.9%) and sedentary individuals (NSCLBP group: 54.8%; control group: 58.1%).

Performance in the 6MST, heart rate responses, and perceived exertion are presented in Table 2. Resting heart rate was similar between groups (∼70 ± 8 bpm). Post‐test heart rate values were higher in the control group for both raters (rater 1: 161 ± 10 bpm; rater 2: 163 ± 10 bpm). The estimated 85% of maximum heart rate was approximately 163 ± 7 bpm for the NSCLBP group and 162 ± 7 bpm for controls. More than 50% of control individuals reached ≥ 85% of maximum heart rate (rater 1: 61.7%; rater 2: 58.1%), whereas in the NSCLBP group, 45.2% reached this threshold in rater 1 and only 22.6% in rater 2.

**TABLE 2:** Six‐minute step test performance, heart rate, and perceived exertion.

Variable	CLBP (rater 1)	CLBP (rater 2)	Control (rater 1)	Control (rater 2)
Six‐minute step test (steps, mean ± SD)				
Total steps (overall)	152 ± 21	160 ± 20	189 ± 18	193 ± 18
Female	149 ± 17	157 ± 15	179 ± 8	183 ± 8
Male	156 ± 26	164 ± 27	204 ± 19	208 ± 18
Heart rate (bpm, mean ± SD)				
Resting heart rate	70 ± 10	70 ± 8	71 ± 8	68 ± 6
Final heart rate	153 ± 10	158 ± 10	161 ± 10	163 ± 10
85% of predicted HRmax	163 ± 7	162 ± 7	—	—
Achieved HRmax thresholds, *n* (%)				
≥ 85% HRmax	14 (45.2)	7 (22.6)	21 (61.7)	18 (58.1)
80%–84% HRmax	10 (32.3)	13 (41.9)	9 (29.0)	11 (35.5)
≤ 80% HRmax	7 (22.6)	11 (35.5)	1 (3.2)	2 (6.5)
Perceived exertion (Borg CR10), *n* (%)				
Easy (2)	—	—	1 (3.2)	—
Moderate (3)	3 (9.7)	2 (6.5)	2 (6.5)	2 (6.5)
Somewhat hard (4)	3 (9.7)	2 (6.5)	—	2 (6.5)
Hard (5–6)	8 (25.8)	8 (25.8)	15 (48.4)	7 (22.6)
Very hard (7–9)	12 (38.7)	13 (41.9)	11 (35.5)	18 (58.1)
Maximal (10)	5 (16.1)	6 (19.4)	2 (6.5)	2 (6.5)

Abbreviations: %: percentage; 6MST: six‐minute step test; CLBP: non‐specific chronic low back pain group; HR: heart rate; HRmax: maximum heart rate; *n*: number of individuals; SD: standard deviation.

Perceived exertion (Borg CR10) increased in the second assessment for both groups. In the NSCLBP group, a greater proportion of individuals rated the test as “very difficult” (38.7% rater 1; 41.9% rater 2) or “maximal effort” (16.1% rater 1; 19.4% rater 2). In the control group, perceived exertion was predominantly classified as “difficult” to “very difficult,” with only two individuals reporting maximal effort in both assessments.

### 3.1 Reliability Analysis

Inter‐rater reliability results are presented in Table 3. The 6MST demonstrated excellent inter‐rater reproducibility. For absolute agreement, intraclass correlation coefficients were 0.915 (95% CI: 0.83–0.96) for the NSCLBP group and 0.973 (95% CI: 0.94–0.99) for the control group, indicating excellent inter‐rater reliability. Relative consistency values were even higher (ICC = 0.979, 95% CI: 0.957–0.990, and ICC = 0.996, 95% CI: 0.992–0.998, respectively), further supporting the consistency of the measurements across raters.

**TABLE 3:** Inter‐rater reliability of the six‐minute step test (rater 1 vs. rater 2).

Group	Reliability	ICC	95% CI	*p*‐value	SEM	MDC
CLBP	Absolute agreement	0.915	0.83–0.96	0.020	6	17
Control	Absolute agreement	0.973	0.94–0.99	0.013	3	8
CLBP	Relative consistency	0.979	0.957–0.990	0.001	3	8
Control	Relative consistency	0.996	0.992–0.998	0.001	1	3

Abbreviations: 6MST: six‐minute step test; CI: confidence interval; CLBP: non‐specific chronic low back pain group; ICC: intraclass correlation coefficient; MDC: minimal detectable change; SEM: standard error of measurement.

The standard error of measurement was 6 steps for the NSCLBP group and 3 steps for controls, while the minimal detectable change was 17 and 8 steps, respectively, indicating acceptable measurement precision. Bland–Altman analysis also demonstrated good agreement between the raters. In the NSCLBP group, the mean bias was 8 steps (SD = 7.7), with limits of agreement ranging from 0.5 to 15.9 steps. Although a weak correlation was observed between means and differences (*r* = 0.129), it was not statistically significant (*p* = 0.488), suggesting no evidence of proportional bias. The slightly higher values observed in the second assessment may reflect a learning or familiarization effect.

In the control group, the mean bias was 4 steps, with limits of agreement ranging from 1 to 6.9 steps. Likewise, no significant association between measurement differences and means was observed (*r* = 0.172, *p* = 0.354), indicating the absence of systematic measurement error (Figure 1).

### 3.2 Construct Validity

Construct validity results are presented in Table 4. Assumptions of normality and homogeneity of variance were met (*p* > 0.05), allowing the use of independent *t*‐tests, except for perceived exertion in rater 2, which was analyzed using the Mann–Whitney test.

**TABLE 4:** Construct validity of the six‐minute step test (known‐groups comparison).

Variable	Rater	*p*‐value	Mean difference (Control–NSCLBP)	SE	Cohen's d
6MST	1	< 0.001^a^	37	5	1.91
	2	< 0.001^a^	33	5	1.76
Resting heart rate	1	0.665^a^	1	2	0.11
	2	0.392^a^	2	2	0.21
Final heart rate	1	0.002^a^	8	3	0.83
	2	0.058^a^	5	2	0.51
Perceived exertion (Borg CR10)	1	0.835^a^	0.09	0.5	0.05
	2	0.483^b^	0.00	0	0.10

*Note:* Data are presented as comparisons between the control and NSCLBP groups for each rater independently. Mean difference represents Control minus NSCLBP; therefore, positive values indicate higher values in the control group. *p*‐values refer to between‐group comparisons (independent *t*‐test or Mann–Whitney test, as appropriate). Cohen's *d* represents the standardized effect size, interpreted as small (0.2), medium (0.5), and large (≥ 0.8).

Abbreviations: 6MST: six‐minute step test; HR: heart rate; NSCLBP: non‐specific chronic low back pain; SE: standard error of the mean difference.

^a^ Independent *t*‐test.

^b^ Mann–Whitney test.

The control group completed significantly more steps than the NSCLBP group in both assessments (both *p* < 0.001), with mean differences of 37 steps for rater 1 and 33 steps for rater 2. These differences were accompanied by very large effect sizes (Cohen's *d* = 1.91 and 1.76, respectively), providing evidence in support of the construct validity of the 6MST for distinguishing between the study groups.

No significant between‐group differences were observed for resting heart rate (Cohen's *d* = 0.11 and 0.21 for raters 1 and 2, respectively) or perceived exertion (Cohen's *d* = 0.05 and 0.10, respectively). Final heart rate was significantly higher in the control group during the first assessment (mean difference = 8 bpm, *p* = 0.002; Cohen's *d* = 0.83), whereas the difference observed during the second assessment (mean difference = 5 bpm) did not reach statistical significance (*p* = 0.058), despite a moderate effect size (Cohen's *d* = 0.51).

### 3.3 Secondary Findings

No significant differences were observed in resting heart rate. Final heart rate was higher in the control group during the first assessment, whereas only a non‐significant trend was observed during the second assessment. Perceived exertion did not differ significantly between groups despite the substantially lower stepping performance observed in individuals with NSCLBP, suggesting a dissociation between subjective effort and objective stepping performance.

### 3.4 Pain, Disability, Catastrophizing, and Kinesiophobia

Pain intensity increased after the 6MST in both assessments. In rater 1, the proportion of individuals reporting mild pain decreased from 74.2% to 32.3%, while moderate pain increased from 25.8% to 45.2%, and severe pain emerged in 22.6% of individuals. Similarly, in rater 2, mild pain decreased from 87.1% to 35.5%, whereas moderate pain increased from 12.9% to 45.2%, and severe pain was reported by 19.4% of individuals after the test. These changes were statistically significant in both assessments (*p* = 0.001), indicating that the test elicited a meaningful increase in pain intensity.

Regarding disability, the majority scored 4 points on the Roland–Morris Disability Questionnaire (51.6%), followed by scores between 5 and 9 (32.3%). Only 9.7% of individuals scored between 10% and 14%, and 6.5% presented scores ≥ 15, indicating higher levels of disability.

Pain catastrophizing levels were moderate, with mean scores of 2.26 ± 0.89 in females and 2.14 ± 1.07 in males, resulting in an overall mean of 2.21 ± 0.95. Kinesiophobia scores were relatively high, with mean values of 41.26 ± 7.7 in females and 39.50 ± 6.2 in males, and an overall mean of 40.58 ± 7.08, suggesting a substantial fear of movement in the sample.

## 4 Discussion

Although the NSCLBP group had a slightly higher body mass index, with 13% classified as obese, both groups had a similar prevalence of being overweight, especially in the control group (64.5%). Previous studies have reported an association between excess body weight and NSCLBP due to increased mechanical load on the lumbopelvic region (Rosa and Felício 2022). However, in the present study, differences in test performance were not explained by body mass index. Similarly, habitual physical activity levels were comparable between the two groups.

Perceived exertion differed between groups. Individuals with NSCLBP more frequently classified the test as “very intense” or “maximal effort,” whereas control individuals predominantly reported “hard” to “very hard” effort levels. This pattern is consistent with previous findings showing that individuals with functional limitations report higher perceived exertion during submaximal tasks (Ricci et al. 2019).

Pain intensity increased following the test in both groups, with a more pronounced shift toward moderate and severe pain levels in individuals with NSCLBP. This response reinforces the test's capacity to provoke symptoms and capture clinically relevant limitations. Similar findings have been reported in studies demonstrating that repetitive physical tasks such as the 6MST may exacerbate symptoms in individuals with chronic pain (Bennett et al. 2016). Previous research has also highlighted the lack of validated functional tools for this population, further emphasizing the relevance of the present findings.

Despite low levels of disability as measured by the Roland–Morris questionnaire, the NSCLBP group exhibited elevated levels of psychological factors such as kinesiophobia and pain catastrophizing. These findings align with evidence suggesting that fear‐avoidance beliefs and maladaptive pain perceptions play a critical role in functional limitation (Da Costa et al. 2017; Van Abbema et al. 2011). Therefore, self‐reported disability measures alone may not fully capture impairment in this population.

Performance in the 6MST clearly differentiated the groups. The control group achieved mean values of 189 ± 18 and 193 ± 18 steps, while the NSCLBP group achieved 152 ± 21 and 160 ± 20 steps. This difference of over 30 steps demonstrates substantially reduced stepping performance. These results are comparable to normative data in healthy adults (Arcuri et al. 2016), supporting the external validity of the control group and reinforcing the test's discriminative capacity. Additionally, although previous studies in other populations have reported associations between 6MST performance and physiological variables such as peak oxygen uptake, such relationships were not evaluated in the present study and should not be inferred from our finding (Ritt et al. 2021).

The reliability analysis demonstrated excellent inter‐rater agreement, with intraclass correlation coefficients exceeding 0.90 for both groups. These values are higher than those reported in earlier studies comparing step and walking tests and are consistent with studies demonstrating the test's high reliability in healthy populations (Salles Albuquerque et al. 2022; Arcuri et al. 2016).

The 6MST's ability to detect changes that exceed measurement error was supported by low standard error of measurement and minimal detectable change values. This indicates that the test provides stable and reproducible estimates of performance across raters. However, these metrics should not be interpreted as evidence of clinically meaningful change since the minimal clinically important difference for the 6MST has not yet been established for individuals with NSCLBP. Future longitudinal studies should investigate the test's responsiveness and determine meaningful clinical improvement thresholds (Weir 2005). These findings are consistent with methodological recommendations and comparable to previous reports (Salles Albuquerque et al. 2022; Da Costa et al. 2017).

Bland–Altman analysis further confirmed good agreement between the raters. In the NSCLBP group, a small mean bias was observed, likely due to a familiarization effect rather than measurement error. Although no familiarization trial was performed, the small, systematic increase observed during the second assessment may reflect increased confidence in the stepping task or optimization of the pacing strategy, rather than reflecting a meaningful change in the participant's underlying status (Salles Albuquerque et al. 2022). This interpretation is exploratory and requires confirmation in future studies designed specifically to evaluate learning effects. The control group showed even narrower limits of agreement, which reinforces the stability of the measurements. These findings align with established methodological standards and support the robustness of the test (Bland and Altman 1986; Sandberg et al. 2020).

Construct validity was confirmed by significant group differences, with large effect sizes observed in both assessments. These differences suggest that the 6MST can capture impairments in stepping performance associated with NSCLBP beyond random variation. The observed between‐group differences align with the expectation that tasks involving repeated lower‐limb loading and lumbopelvic stabilization place greater demands on individuals with pain‐related movement limitations (Souza et al. 2017).

To our knowledge, this is the first study to propose using the 6MST to assess stepping performance in individuals with NSCLBP. By demonstrating reliability and validity, this study fills an important gap in the literature and provides a practical, low‐cost, clinically applicable tool for evaluating stepping performance in this population.

While the 6MST demonstrated excellent measurement properties in this study, our findings should not be interpreted as evidence that it is superior to other established, field‐based functional assessments, such as the six‐minute walk test. Rather, the available evidence suggests that these instruments may provide complementary information depending on the clinical context, available space, and functional demands of interest. Further comparative studies evaluating responsiveness, prognostic value, and patient acceptability are needed to better define the role of the 6MST in clinical practice.

Some limitations should be acknowledged. First, the inter‐rater reliability design involved different examiners evaluating participants on separate occasions, 7 days apart. Consequently, the observed agreement may reflect examiner consistency, as well as potential familiarization or adaptation effects. Additionally, no familiarization trial was performed before testing, which may have contributed to the small systematic differences observed between assessments. Second, the sample consisted predominantly of young adults with relatively low disability levels. Participants were recruited through university advertisements and social media, which may have introduced selection bias toward community‐dwelling individuals with a greater willingness to participate in research.

Therefore, the findings should be generalized with caution, particularly to older adults and individuals with more severe functional impairment. Third, responsiveness and the minimal clinically important difference were not evaluated, which prevents conclusions from being drawn about the test's ability to detect clinically meaningful improvements over time. Fourth, construct validity was assessed using a known‐groups approach. While this approach is accepted for demonstrating construct validity, additional convergent validity analyses examining the associations between the 6MST and measures of disability, pain intensity, kinesiophobia, pain catastrophizing, and physical activity would strengthen the psychometric evidence supporting the instrument.

In conclusion, the findings of this study demonstrate that the 6MST provides reliable and valid measurements of stepping performance in individuals with NSCLBP. The test showed excellent inter‐rater reliability and strong agreement between raters and successfully discriminated between individuals with and without NSCLBP, with large effect sizes observed across assessments.

Compared with healthy controls, individuals with NSCLBP completed fewer steps, were less likely to reach the predefined target heart rate during the test, and experienced greater increases in pain intensity following the assessment despite reporting similar levels of perceived exertion. These findings indicate that the 6MST can detect meaningful differences in observed stepping performance and test responses between groups but should not be interpreted as evidence of differences in exercise tolerance, aerobic capacity, or cardiopulmonary function.

Due to its practicality, low cost, and ease of administration, the 6MST may be a useful performance‐based tool for evaluating stepping performance in individuals with NSCLBP. Future longitudinal studies should investigate its responsiveness, establish its minimal clinically important difference, and compare its measurement properties with those of other established functional assessments.

## 5 Implications for Physiotherapy Practice

- The 6MST showed excellent inter‐rater reliability (ICC > 0.90) and minimal measurement error.
- The test clearly distinguished between groups that differed by more than 30 steps, with very large effect sizes (*d* > 1.7).
- Individuals with NSCLBP exhibited significantly poorer stepping performance and greater post‐test pain.

## Author Contributions

All authors (Francisco Basílio da Silva‐Júnior, André Pontes‐Silva, ⁠Yane Pelicer‐Marques, Sabrina Marinho Coutinho, Marco Fabrício Dias‐Peixoto, Kassiana de Araujo Pessôa, Andréa Dias Reis, Cristiano Teixeira Mostarda, Almir Vieira Dibai‐Filho, Flávio de Oliveira Pires) – conceptualization, data curation, formal analysis, investigation, methodology, validation, visualization, writing (original draft, review, and editing). Prof. André Pontes‐Silva was responsible for translating and submitting the article, as well as handling further editorial procedures.

## Funding

The Article Processing Charge (APC) for the publication of this research was funded by the Coordenação de Aperfeiçoamento de Pessoal de Nível Superior – Brasil (CAPES) (ROR identifier: 00x0ma614). Study was funded by the CAPES (code 001). The funding source had no role in the study design, collection, analysis, interpretation of data, writing of the report, or in the decision to submit the article for publication.

## Ethics Statement

Study was approved by the Research Ethics Committee of the Universidade Federal do Maranhão, Sao Luís, MA, Brazil (report number: 5.404.643).

## Consent

Informed consent was obtained from all subjects and/or their legal guardian(s). All respondents participated in this study freely and with their consent. All experiments were conducted in accordance with the tenets of the Declaration of Helsinki.

## Conflicts of Interest

Professor Dr. André Pontes‐Silva is a reviewer for the Physiotherapy Research International. The author declares no further competing interests.

## Data Availability

The data and materials in this paper are available from the corresponding author on request.
